# Assessment of Tunnel Lining Stability through Integrated Monitoring of Fiber Bragg Grating Strain and Structural Deformation

**DOI:** 10.3390/s24123824

**Published:** 2024-06-13

**Authors:** Chuan Li, Dechao He, Jiaqi Li, Qiang Xu, Xiaorong Wan, Jianning Su

**Affiliations:** 1 Faculty of Information Engineering and Automation, Kunming University of Science and Technology, Kunming 650500, China; lichuan@kust.edu.cn (C.L.); 20222204249@stu.kust.edu.cn (D.H.); ljqkust@stu.kust.edu.cn (J.L.); 2 Yunnan Key Laboratory of Computer Technology Applications, Kunming 650500, China; 3 Yunnan Aerospace Engineering Geophysical Detecting Co., Ltd., Kunming 650200, China; xuq@aerospace.net.cn (Q.X.); sujn@aerospace.net.cn (J.S.)

**Keywords:** tunnel lining, structural deformation, strain monitoring, stress–strain, thermal strain, fiber Bragg grating strain sensor, stability

## Abstract

Tunnel excavation induces the stress redistribution of the surrounding rock. Structural cracks may develop in the secondary lining due to this stress redistribution and bias pressure, consequently affecting the overall construction safety of the tunnel. This paper aims to achieve real-time monitoring of the excavation stability of the lining structure by integrating two monitoring technologies: structural deformation monitoring and fiber grating strain monitoring. Additionally, it proposes a method to simultaneously measure the thermal strain and applied stress–strain of the structure. By analyzing the displacement and deformation of the lining structure, its stability can be preliminarily evaluated in the short term. To achieve long-term real-time monitoring and a more accurate assessment of the tunnel structure’s stability, the paper introduces fiber Bragg grating (FBG) strain sensor monitoring technology. First, based on the geological stratigraphy information obtained from the exploration, a simulation model of the tunnel under different section bias angles is established. The displacement and stress concentration areas of the lining structure are then analyzed to optimize the sensor deployment array and provide a theoretical basis for the sensor arrangement. FBG strain sensors are installed on the surface of the structure to measure thermal strain and loading stress–strain, whereas FBG temperature sensors measure local temperature. The findings indicate that following tunnel excavation, the maximum daily strain differences at K107+043 and K107+240 were 126.87 µε and 209.38 µε, respectively. After a period of rock disturbance, the average daily strain differences due to applied stress–strain were 16.8 µε and 12.65 µε, respectively. The thermal strain was close to the daily strain difference. Therefore, after the rock disturbance subsided, the strain fluctuations in the lining structure were mainly caused by local temperature changes, and the surrounding rock tended to stabilize. This offers a viable method for evaluating structural stability post-tunnel excavation.

## 1. Introduction

During tunnel excavation, stress redistribution in the surrounding rock occurs. If the redistributed stress exceeds the elastic range of the surrounding rock and surpasses the axial compressive or tensile strength of the concrete lining, the lining will crack, affecting the structural health of the tunnel [1]. Monitoring tunnel safety and stability requires first determining the settlement deformation in the lining structure due to stress redistribution [2,3]. Some scholars have found through finite element simulations, tunnel scale models, and actual engineering studies that stress redistribution mainly results in settlement deformation and strain changes in the lining structure [4,5,6,7,8].

In conjunction with an analysis of tunnel settlement deformation, the subsequent scholars employed diverse monitoring and analysis techniques to investigate the convergence deformations in tunnel settlement throughout the project. Li et al. [9] established monitoring points for transverse horizontal convergence and vault settlement within the Nanjing Metro shield tunnel. Subsequent to data processing, a comprehensive qualitative and quantitative evaluation of the deformation state of either the entire or specific segments of the monitored structure is conducted. Guo et al. [10] investigated the stress characteristics of the tunnel lining in the significantly deformed section across various construction stages. This was achieved by monitoring the displacement of the carbonaceous slate surrounding rock, as well as the internal forces within both the primary and secondary linings throughout the entire construction phase of the Haibalo Tunnel on the Xiangli Expressway. The vault settlement value is monitored in areas exhibiting significant lining deformation. Subsequently, the safety and stability of the tunnel lining structure during excavation are analyzed by considering both the displacement of surrounding rock and the settlement of the vault. Zhao et al. [11] employed a construction monitoring approach to analyze the settlement and horizontal deformation traits of an existing tunnel, utilizing the excavation of the Yangtaishan Tunnel on Shenzhen Metro Line 6 as an illustrative engineering case study. This analysis determined the distribution of tunnel excavation lining deformations, alongside evaluating the stability of the lining structure across various stages of horizontal deformation and vault settlement deformation.

The stability of tunnel engineering can be assessed by monitoring the strain variations in the secondary lining. Various types of sensors are used for strain monitoring, including resistive strain gauges, micro-electro-mechanical system (MEMS) sensors, and fiber-optic sensors, etc. Due to the different monitoring ranges and point continuity, fiber optic sensors can be classified as pointwise sensors (where the sensing elements of the system are separate parts) and as distributed ones. These sensors primarily use optical fibers. Distributed sensing technologies, such as Optical Frequency Domain Reflectometry (OFDR) and Phase-Sensitive Optical Time Domain Reflectometry (ϕ-OTDR), enable global strain measurements on a single fiber [12,13]. Xu et al. [14] obtained the spatiotemporal evolution characteristics of cracks in soil strain fields using OFDR distributed strain measurement, establishing the correlation between strain and cracks. Liu et al. [15] analyzed concrete cracking behavior using a distributed OFDR fiber optic sensor network. Their results revealed the relationship between concrete crack bond stress and slip behavior. Single-point fiber optic measurement typically employs fiber Bragg grating (FBG) strain sensors, offering flexible installation options. Each FBG sensor corresponds to a specific location, and multiple sensors can be connected in series for multipoint measurements. In structures requiring localized measurements, FBG sensors are cost-effective, easy to install and maintain, and provide fast response to signals. The FBG sensor possesses characteristics including a wide measurement range, minimal transmission loss, high accuracy, and reliable operation even in challenging environments. This makes it well-suited for the real-time monitoring of strain and temperature in large-scale structures [16,17,18]. It has been widely used in the field of engineering monitoring. In tunnel engineering, the rational arrangement of sensors can more accurately monitor the stability of the structure. Zhu et al. [19] deployed seven strain gauges equidistantly along the circumferential section of a mountainous tunnel under complex geological conditions to measure structural strain and analyze construction safety. Ma et al. [20] used fully distributed fiber optic sensing technology, laying four optical cables from the entrance to the exit of the tunnel. These cables were positioned directly above, below, east, and west of the shield tunnel to measure structural strain and analyze tunnel convergence deformation. Li et al. [21] affixed a differential FBG strain sensor onto the surface of the secondary lining within the Shanxinpo Tunnel, acquiring both strain and temperature data for the secondary lining. Simultaneously, an analysis of the primary reasons for secondary lining strain during backfilling and operation was conducted, drawing upon the thermal strain of the lining as a reference. Li et al. [22] employed fiber grating strain sensors and temperature sensors to concurrently measure both the apparent thermal strain and applied stress–strain of the tunnel’s secondary lining structure amidst complex conditions. They conducted an analysis of frost heaving effects on the surrounding rock of the tunnel, based on variations in applied stress and strain. Zhu et al. [23] proposed employing the “correlation coefficient method” to accurately separate load strain from thermal strain in strain monitoring data. This method entails determining the temperature effect correction coefficient within the theoretical formula. Analysis results indicate that thermal strain exhibits variations corresponding to external temperature changes throughout the monitoring period. Notably, thermal strain demonstrates a discernible upward trend, whereas load strain remains relatively stable, fluctuating within a specific range.

Throughout the tunnel excavation process, the settlement deformation of the lining structure is influenced by stress redistribution within the surrounding rock. The pressure exerted by the surrounding rock on the lining structure results in deformation. This study integrates structural deformation analysis with fiber Bragg grating strain monitoring to assess tunnel structure stability. ABAQUS simulation (ABAQUS 6.17) is used to analyze the deformation and stress characteristics of the secondary lining. Additionally, horizontal convergence and vault settlement values are monitored. Displacement is monitored based on simulation analysis, with abnormal points selected for monitoring sections. Fiber grating sensors are deployed to obtain applied stress–strain and thermal strain data of the secondary lining. The changing patterns of these factors during the excavation process are analyzed, offering insights for safety and stability evaluation during tunnel excavation.

## 2. Structural Deformation and Stress Analysis of Tunnel Lining Section

**Figure 1:**
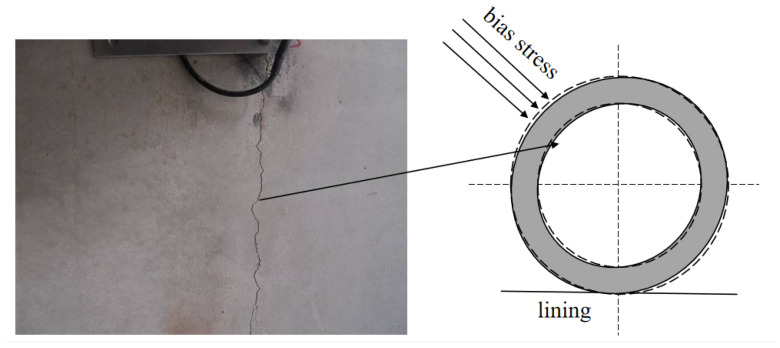
Deformation damage of tunnel lining structure.

The Deqin Tunnel is located in the high-altitude area of western Yunnan, China. The altitude of the tunnel site area is 3416.68–3662.00 m. The overall length of the tunnel is 1530 m; the maximum depth of the tunnel is approximately 242.47 m. The tunnel body is on a slope along the vertical direction of the tunnel, with a slope of 15 to 35°. The slope section is asymmetrical along the axis of the cave body, and there is a bias phenomenon. During the tunnel excavation process, the eccentric pressure of the surrounding rock causes the tensile stress of the lining structure to exceed the bearing range of the lining structure, and the side walls of the structure shrink and deform. When the maximum stress on the side walls exceeds the maximum load-bearing range of the concrete, the lining structure has structural instability and cracking [24]. The cracking of the lining structure is shown in Figure 1.

**Figure 2:**
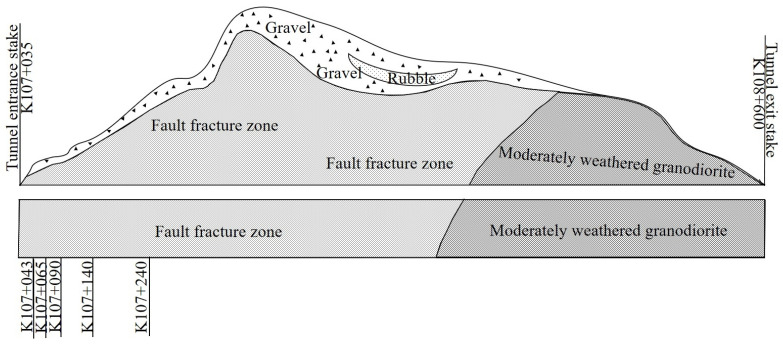
Geological longitudinal section of Deqin Tunnel.

Based on findings from engineering geological surveys and geophysical explorations, it is determined that the entire tunnel site area is covered by a sparse layer of Quaternary Holocene slope gravel. Predominantly comprising slate and shale, the entrance and tunnel body of the site give way to granodiorite at the exit section. The slate and shale rock layers exhibit an angle of inclination measuring 60 degrees. Based on measurements and statistical analysis of bedrock outcrops at the site, it is evident that the rock mass within the tunnel site area exhibits structural joints and relatively extensive fissures. The tunnel traverses primarily through the fault fracture zone comprised of slate and shale, transitioning to the granodiorite layer at the exit section. Figure 2 displays the longitudinal section of the tunnel.

To determine the optimal sensor installation location and ensure reliable monitoring data, the displacement and stress fields of the tunnel’s secondary lining were carefully examined through finite element analysis based on the unique geological properties of the Deqin Tunnel. According to Figure 2, the K107+065 section was selected to establish a two-dimensional model of the tunnel section. Since the mountain’s inclination along the vertical direction of the tunnel is between 15° and 35°, and the bias angles of each tunnel section vary, the structural deformation and stress of the tunnel lining structure were analyzed at deflection angles of 15°, 25°, and 35°, so as to optimize the sensor layout points for internal measurement of the tunnel lining structure. The simulation parameters are shown in Table 1.

**Figure 3:**
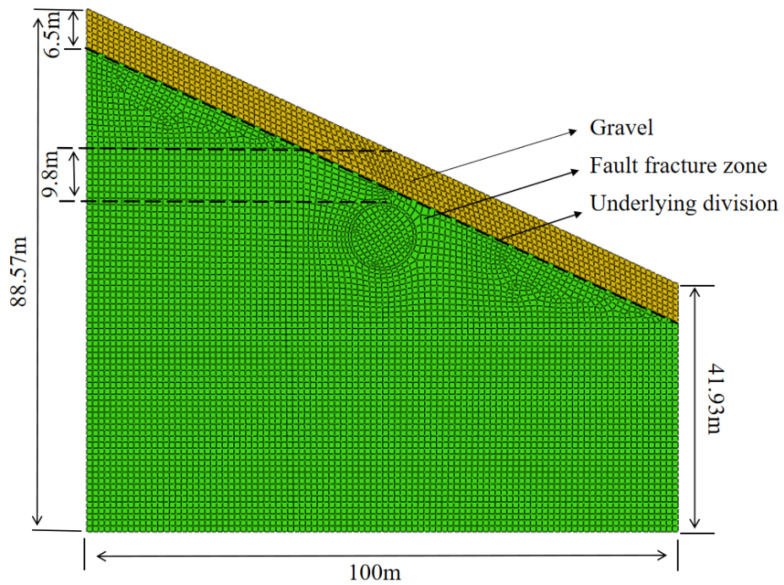
Schematic diagram of tunnel cross-section model.

Assume stress and strain variations across all materials remain within the elastic-plastic threshold, independent of spatial influences and moisture content. The tunnel structure is influenced solely by the gravitational forces exerted by the surrounding rock mass. Considering the actual depth and dimensions of the tunnel, the model’s width is established at 100 m. At a burial depth of 9.8 m, the left side heights of the three biased models are 71.84 m, 88.57 m, and 100.26 m, the heights on the right side are 51.85 m, 41.93 m, and 30.24 m, respectively, corresponding to bias angles of 15°, 25°, and 35°. There are two strata in the mountain from top to bottom: the gravel layer and the fault fracture zone composed of slate and shale. The gravel layer has a fixed height of 6.5 m. Throughout the tunnel excavation process, the upper boundary corresponds to the free surface, with horizontal displacement constraints applied to the boundaries flanking the mountain, whereas the bottom boundary remains fixed. The initial stress field only considers the gravity stress field and ignores the tectonic stress field. Through the establishment of corresponding construction stages, the simulation of the tunnel construction process is finalized. The primary objective is to streamline the dynamic excavation process into a methodical static construction sequence, achieved through two distinct approaches: “activation” and “freezing”. Throughout the excavation process, the soil slated for removal undergoes freezing, whereas support and lining units are activated, culminating in the completion of the tunnel excavation simulation. Figure 3 illustrates the cross-sectional model of the tunnel with a 25° bias.

**Figure 4:**
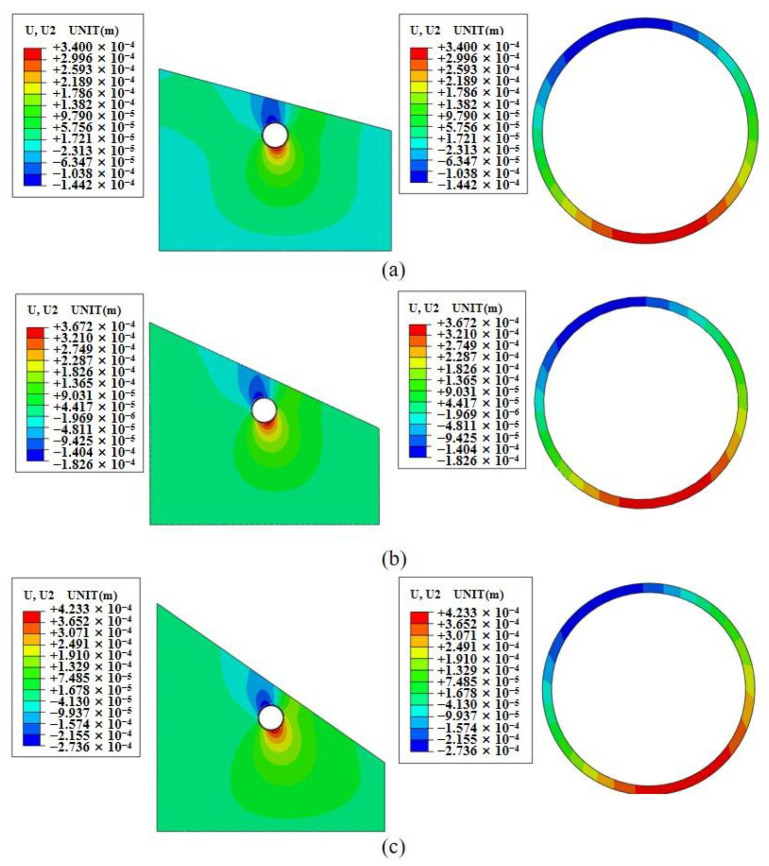
The Y-direction displacements of three types of tunnels under bias angles. (**a**) 15° bias, (**b**) 25° bias, (**c**) 35° bias.

Figure 4 illustrates the displacement and deformation of the tunnel under biased pressure. Deformation is mainly concentrated at the vault and invert locations. In Figure 4a, with a 15° bias angle, the vault experiences a maximum settlement of 0.14 mm, and the invert arch reaches a maximum uplift of 0.34 mm. In Figure 4b, with a 25° bias angle, the vault settles by up to 0.19 mm, and the invert arch lifts by up to 0.37 mm. Figure 4c shows vertical deformation under a 35° bias angle, with the invert arch lifting by up to 0.42 mm and the vault settling by up to 0.27 mm. This analysis shows that as the bias angle increases, the displacement and deformation of vault settlement and invert uplift also increase. The focal point of displacement and deformation shifts from the vault to the spandrel as the bias angle increases. Therefore, the concentration point of displacement and deformation moves towards the bias direction as the bias angle increases.

**Figure 5:**
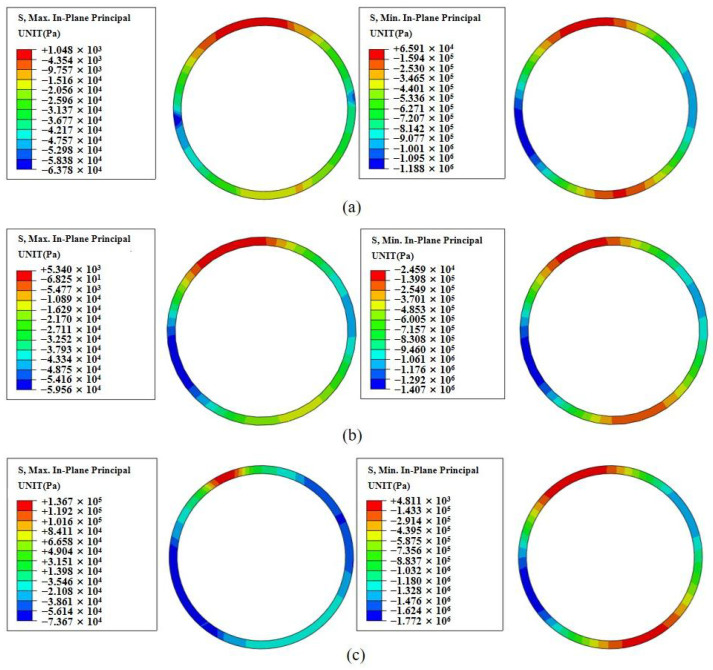
Stress distribution of tunnel lining under three bias angles. (**a**) 15° bias, (**b**) 25° bias, (**c**) 35° bias.

Figure 5 illustrates the simulation-based analysis of stress characteristics observed in the secondary lining post-tunnel excavation across varying bias angles within the tunnel section. The secondary lining has the function of controlling the deformation of the surrounding rock to support the safety of the tunnel structure. Observations from Figure 5 reveal that stress concentration within the tunnel primarily manifests as tensile stress at the vault and inverted arch, alongside compressive stress at the bottom of the arch on both sides. The predominant cause of tensile stress stems from the displacement occurring from the higher side to the lower side due to the mountain’s bias angle. Concomitantly, the upper portion converges towards the axial direction of the tunnel, whereas compressive stress arises due to the stress exerted by the biased surrounding rock. The lining structure undergoes compression in alignment with the stress direction of the surrounding rock, resulting in the expansion and outward compression of the arch feet on both sides. In Figure 5a, the stress distribution under a 15° bias angle shows a maximum tensile stress of 0.001 MPa at the arch apex and a maximum compressive stress of 1.19 MPa at the left arch base. Figure 5b shows a 25° bias angle, with the maximum tensile stress of 0.005 MPa at the arch apex and the maximum compressive stress of 1.43 MPa at the left arch base. Figure 5c shows a 35° bias angle, with the maximum tensile stress of 0.14 MPa at the arch apex and the maximum compressive stress of 1.77 MPa at the left arch base. With an increase in the bias angle, both the tensile and compressive stresses on the lining structure escalate. As the bias angle increases, the region of tensile stress concentration at the vault shifts towards the arch in the biased direction. Additionally, with the increasing bias angle, the compressive stress concentration area at the arch foot shifts towards the arch waist. Notably, the magnitude of the offset amplifies with a larger bias angle.

In tunnel engineering, if there is no bias from the mountain, the inner side of the tunnel lining at the arch crown generally experiences the maximum displacement and the greatest tensile stress, whereas the inner sides of the arch waists on both sides of the lining bear the maximum compressive stress. In the presence of bias, the areas of maximum displacement and tensile stress on the inner side of the tunnel lining at the arch crown shift towards the side of the bias. Similarly, the areas of maximum compressive stress on the inner sides of the arch waists shift circumferentially towards the side of the bias. Upon analysis of Figure 4 and Figure 5, it is evident that under left-sided bias angles of 15°, 25°, and 35°, the areas of maximum tensile stress and maximum displacement in the lining structure shift approximately 13°, 22°, and 30° from the arch crown circumferentially to the left, respectively. The areas of maximum compressive stress in the lining structure also shift approximately 13°, 22°, and 30° from the arch waist circumferentially to the left, respectively. Therefore, to more accurately assess the stability of the tunnel, the optimal sensor installation positions are at the arch crown, the arch shoulders shifted 30° circumferentially to both the left and right from the arch crown, the arch waists on both sides, and the arch bases on both sides shifted 30° circumferentially to both the left and right from the arch waists.

## 3. Joint Monitoring of Structural Deformation and Strain of Tunnel Lining Structures

Tunnel settlement and peripheral convergence are critical indicators for evaluating the safety and stability of tunnel structures. Initial on-site observations reveal axial compression cracking along the inner wall of the tunnel lining. The displacement of the tunnel support structure reflects the mechanical behavior of the structure. Therefore, stability can be assessed through displacement analysis. To monitor structural deformations, five sections from K107+043 to K107+240 were selected for peripheral convergence and vault settlement monitoring. The vault and peripheral displacements of these sections were tracked over a 21-day period (once daily) to assess their conditions and analyze the monitoring outcomes.

**Figure 6:**
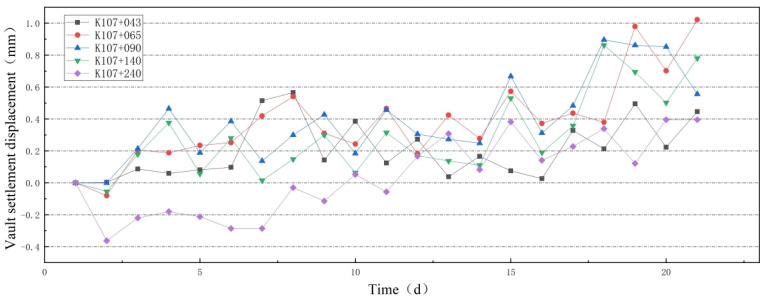
Vault settlement displacement changes with time.

The settlement of the tunnel lining structure is influenced by the surrounding rock, causing variations in vault displacement. During monitoring, a positive vault displacement value indicates downward movement, whereas a negative value indicates upward extrusion deformation. Figure 6 shows the vault settlement displacement.

Analysis of the vault settlement monitoring data reveals a continuous alternation between upward extrusion and downward movement of the tunnel vault throughout the monitoring period. Similar findings were reported in Xie et al.’s [25] investigation on concrete structure damage. Concrete structures subjected to reciprocating loads are more prone to fatigue-induced damage and cracking compared to unidirectional loading. Analysis of the monitoring data indicates that the repetitive up-and-down movement of the vault, resulting from upward squeezing force and downward surrounding rock pressure, can lead to damage and cracking of the concrete lining structure.

**Figure 7:**
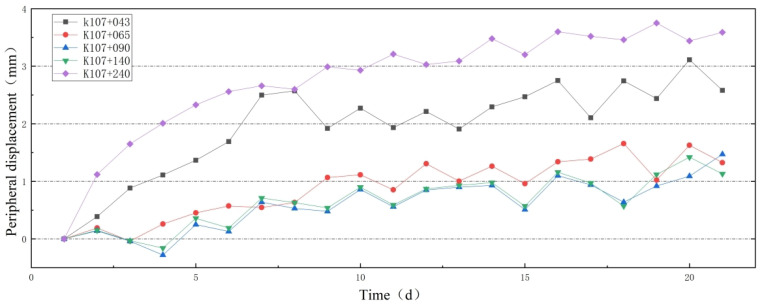
Peripheral convergence displacement changes with time.

Peripheral convergence of the lining structure serves as a crucial indicator of structural deformation. Peripheral displacement refers to the displacement of measuring points on the surrounding rock within the tunnel boundary post-excavation. Accurately measuring the absolute displacement value of a spatial point is challenging [26]. Consequently, determining the relative displacement between two points along the direction of the tunnel’s inner wall is typically adopted. Changes in peripheral displacement are manifested through the displacement between the arch bases on either side of the cavern. In monitoring data, negative values indicate inward convergence, whereas positive values signify outward extension deformation. Figure 7 illustrates the peripheral displacement.

Analysis of the tunnel’s peripheral displacement monitoring data reveals alternating patterns of inward convergence and outward expansion throughout the monitoring period. The maximum daily inward convergence displacement measures 1.12 mm, whereas the maximum daily outward expansion displacement reaches 0.652 mm. The peripheral side walls’ concrete structure is susceptible to damage and cracking due to the reciprocating load exerted by the lining concrete structure.

The vault settlement and peripheral convergence displacement of the lining, influenced by surrounding rock pressure, provide insights into the displacement trends of the lining structure from a macroscopic viewpoint [27], facilitating short-term evaluation of the lining structure’s stability. All five selected monitoring sections exhibited structural instability throughout the monitoring period. Structural deformation results from the accumulation of the structure’s own strain. To enhance the accuracy of evaluating the stability of the tunnel lining structure, strain monitoring of the lining structure is implemented. Strain monitoring of the lining structure is conducted using fiber grating strain sensors, facilitating long-term monitoring and enabling further analysis of structural stability by monitoring structural strain and scrutinizing strain changes from a microscopic perspective.

**Figure 8:**
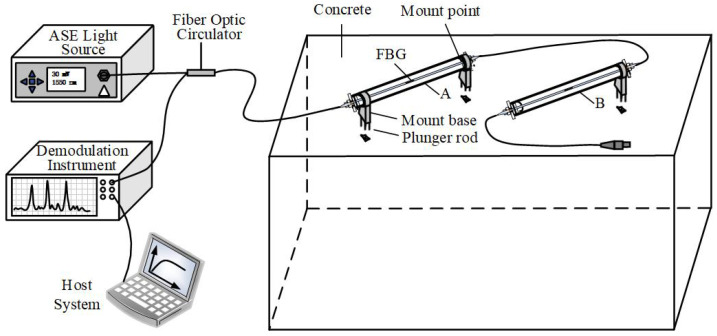
Monitoring principle of fiber grating strain detection system.

Figure 8 illustrates the fiber Bragg grating (FBG) strain measurement system, comprising an ASE broadband light source, an FBG strain sensor, an FBG demodulator, and a host system. The FBG strain sensor is connected via a circulator, and the demodulator analyzes the wavelength reflected by the sensor to determine the total strain and temperature of the structure. The ASE broadband light source has an output power of 10 mW, a spectral flatness of less than 2 dB, and a wavelength range of 1515–1595 nm. The wavelength demodulation accuracy of the optical signal demodulation equipment is 1 pm, the sampling frequency is 2 kHz, and the wavelength repetition rate can be less than 3 pm. The wavelength demodulation range matches the ASE broadband light source. The strain measurement range of the FBG sensor is −3000 to 3000 µε, and the temperature measurement range is −40 °C to 120 °C. The accuracy is within 1 µε, and the wavelength range matches that of the ASE broadband light source. Strain sensor A is affixed to the structure’s mid-surface using anchor rods at both ends, measuring the total strain of the structure. The total strain includes both applied stress–strain and thermal strain. Strain sensor B is affixed to the structure’s mid-surface at one end of the anchor rod, with its wavelength return solely accounting for the temperature-induced offset, used for temperature compensation. The thermal strain of the structure is computed based on the measured temperature, enabling concurrent measurement of applied stress–strain and thermal strain on the structure.

As shown in Figure 8, is the Bragg wavelength shift of sensor A, is the Bragg wavelength shift of sensor B, $\Delta \lambda_{g}$, and $\Delta \lambda_{T}$ can be described as:

(1) $$\left\{\begin{array}{c} \Delta \lambda_{g}=S_{\epsilon }C_{g,\epsilon }\epsilon +\left[S_{T}C_{g,T}+S_{\epsilon }C_{g,\epsilon }C_{g,T}\left(\alpha_{H}-\alpha \right)\right]\Delta T=S_{\epsilon }^{S}\epsilon +\left[S_{T}+S_{\epsilon }^{S}\left(\alpha_{H}-\alpha \right)\right]C_{g,T}\Delta T\\ \Delta \lambda_{T}=\left[S_{T}C_{g,T}+S_{\epsilon }C_{g,\epsilon }C_{g,T}\left(\alpha_{Fe}-\alpha \right)\right]\Delta T=S_{T}^{T}\Delta T \end{array}\right.$$

where ε is the strain change in structure, $\Delta_{T}$ is the temperature variation, $\alpha_{H}$ is the thermal expansion coefficient of the structure and is determined by the physical parameters of the structure, $\alpha_{Fe}$ = 11.8 × $10^{-6}\;{}^{\circ}C^{-1}$ is the thermal expansion coefficient of the steel pipe, α = 0.55 × $10^{-6}\;{}^{\circ}C^{-1}$ is the thermal expansion coefficient of the fiber. $C_{g,\epsilon }$ ≈ 0.85 is the strain transmission coefficient between fiber and host structure, and $C_{g,T}$ ≈ 0.94 is the thermal transmission coefficient of the en-capsulated FBG. At 1550 nm, the strain and thermal sensitivities are $S_{\epsilon }$ ≈ 1.21 pm/με and $S_{T}$ ≈ 10.3 pm°$C^{-1}$ for silica-based fiber Bragg grating. $S_{\varepsilon }^{S}$ = $S_{\varepsilon }C_{g,\varepsilon }$ ≈ 1.03 pm/με is the strain sensitivities of the FBG sensor A anchored on the surface of the structure, $S_{T}^{T}$ = $S_{T}C_{g,T}+S_{\epsilon }C_{g,\epsilon }C_{g,T}\left(\alpha_{Fe}-\alpha \right)$ ≈ 20.56 pm°$C^{-1}$ is the thermal sensitivities of the FBG sensor B suspended on the surface of the structure. According to Equation (1), the temperature variation ΔT and the applied stress–strain ε measured by FBG can be devoted to

(2) $$\left\{\begin{array}{c} \Delta_{T}=\frac{\Delta \lambda_{T}}{S_{T}^{T}}\\ \varepsilon =\frac{\Delta \lambda_{g}}{S_{\varepsilon }^{S}}-\frac{\left[S_{T}+S_{\varepsilon }^{S}\left(\alpha_{H}-\alpha \right)\right]C_{g,T}\Delta \lambda_{T}}{S_{\varepsilon }^{S}S_{T}^{T}} \end{array}\right.$$

where $\varepsilon_{T}$ is the temperature difference expressed approximately as the structure. Therefore, the equivalent thermal strain $\varepsilon_{T}$ and applied stress–strain $\varepsilon_{W}$ of the structure can be devoted as

(3) $$\left\{\begin{array}{c} \varepsilon_{T}=\alpha_{H}\Delta_{T}=\frac{\Delta \lambda_{T}}{S_{T}^{T}}\alpha_{H}\\ \varepsilon_{W}=\varepsilon -\varepsilon_{T}=\frac{\Delta \lambda_{g}}{S_{\varepsilon }^{S}}-\frac{\left[S_{T}C_{g,T}+S_{\varepsilon }^{S}\alpha_{H}\left(C_{g,T}-1\right)-S_{\varepsilon }^{S}C_{g,T}\alpha \right]\Delta \lambda_{T}}{S_{\varepsilon }^{S}S_{T}^{T}} \end{array}\right.$$

Following an analysis of structural deformation, five monitoring sections, spanning from K107+043 to K107+240, were identified for the deployment of FBG sensors. Given that the inward displacement and outward expansion of the lining structure, prompted by the surrounding rocks, induce circumferential strains along the section, a total of seven FBG strain sensors were installed in circumferential positions along the section to measure the strain within the lining structure. Additionally, two FBG strain sensors for measuring lining temperature were strategically placed—one on the vault top and the other on the left arch foot.

**Figure 9:**
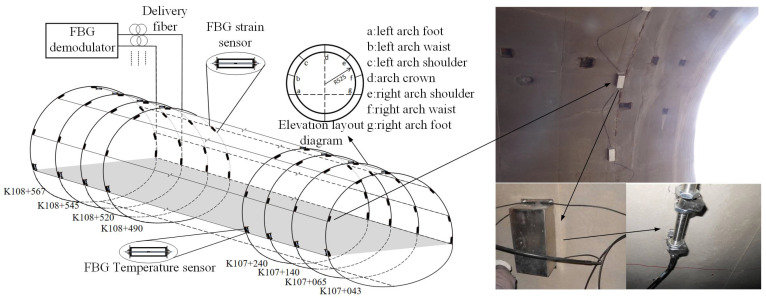
Schematic diagram of fiber Bragg grating sensor layout.

The FBG strain sensor, employed for detecting structural strain, is affixed at two specific points on the anchor rod, integrated within the concrete lining, facilitating the conversion of secondary lining strain into axial strain for the fiber Bragg grating strain sensor. Furthermore, one end of the fiber Bragg grating strain sensor, designated for monitoring the temperature of the secondary lining, is welded onto the anchor rod embedded within the concrete lining. It is noteworthy that this sensor solely focuses on temperature measurement without being subjected to structural strain. The precise location and installation method are illustrated in Figure 9.

Upon the installation of the fiber Bragg grating strain sensor onto the outer surface of the tunnel secondary lining, the detected applied stress–strain and apparent thermal strain on the secondary lining can be mathematically expressed as follows:

(4) $$\left\{\begin{array}{c} \varepsilon_{T}=0.05\alpha_{E}\Delta \lambda_{T}\\ \varepsilon_{W}=0.97\Delta \lambda_{g}-\left(0.432-0.003\alpha_{E}\right)\Delta \lambda_{T} \end{array}\right.$$

Given that the design grade of the tunnel’s secondary lining concrete is C30, let $\alpha_{E}={10\times 10}^{-6}{}^{\circ}C^{-1}$ represent the thermal expansion coefficient of the secondary lining structure. A strain calibration experiment on the FBG strain sensor was performed by applying different loads at both ends of the sensor. According to the experimental data, the least squares method is used to obtain the linear curve of the relationship between strain and wavelength, which can be expressed as λ = 0.001ε + 1544.2. Here, λ represents wavelength (unit: nm), and ε represents microstrain (unit: µε). The strain sensitivity coefficient of the FBG strain sensor is 1.028 pm/µε, the nonlinear error is 0.2%, and the repeatability error is 0.68%. Therefore, the experimental calibration Equation (4) can thus be formulated as follows:

(5) $$\left\{\begin{array}{c} \varepsilon_{T}=0.48\Delta \lambda_{T}\\ \varepsilon_{W}=0.97\Delta \lambda_{g}-0.402\Delta \lambda_{T} \end{array}\right.$$

## 4. Analysis of Monitoring Results of Tunnel Lining Strain

**Figure 10:**
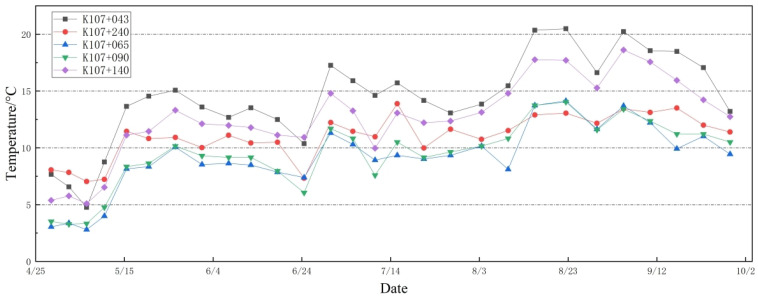
Temperature change curve of each monitoring section.

Through monitoring the tunnel sections, temperature readings from the vaults and the left arch foot of five specific sections were acquired. Considering the negligible temperature discrepancy between two monitoring points on the same road section, the average value of these points is adopted as the representative temperature for each road section. The temperature fluctuations across the five-month period for each section are illustrated in Figure 10. Throughout this time frame, from April to September, there was a gradual increase in temperature across all sections, culminating in a peak before gradually receding. Figure 10 reveals that, commencing from section K107+043 positioned at the tunnel opening, the temperature of the secondary lining gradually diminishes with increasing tunnel depth. This phenomenon can likely be attributed to the heightened influence of the external ambient temperature as the opening nears the outer environment. Notably, the temperature observed at section K107+240 exceeds that of sections K107+065 to K107+140. This discrepancy may stem from the presence of geothermal energy within the cavern. The findings suggest that the temperature near the tunnel entrance is more susceptible to short-term fluctuations in external ambient temperature compared to deeper tunnel regions. Section K107+240 exhibits pronounced temperature fluctuations owing to the combined effects of ambient temperature and geothermal energy.

**Figure 11:**
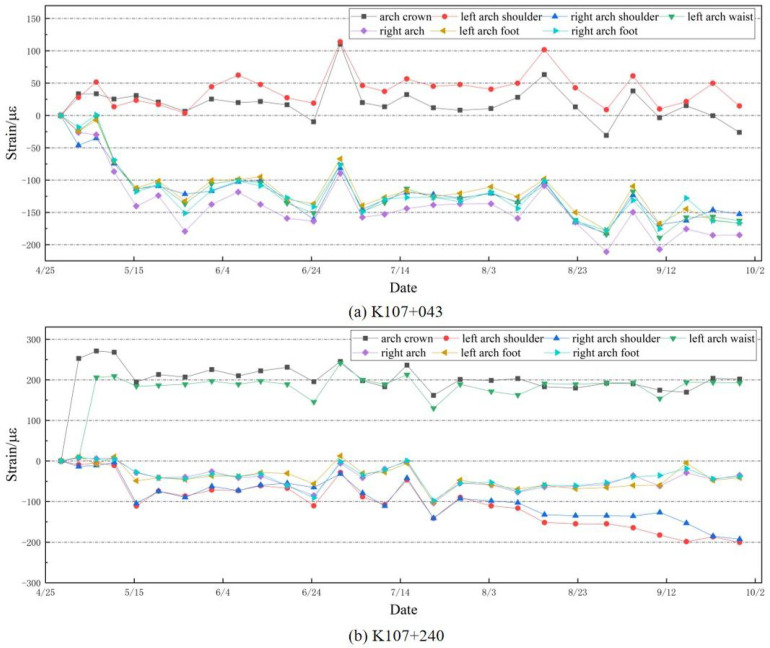
Total strain changes in two sections K107+043 and K107+240 over 5 months.

The total strain in each section was monitored from April to September (twice a week). The detection results of two typical sections, K107+043 and K107+240, are analyzed, as shown in Figure 11. According to Figure 11a, the monitoring section at K107+043 indicates that the maximum compressive stress in the lining structure shifts from the arch crown to the left arch shoulder, with the left arch shoulder experiencing the greatest compressive stress, followed by the arch crown. The maximum compressive stress shifts from the right arch foot to the right arch waist, with the right arch waist experiencing the greatest tensile stress, followed by the right arch foot. Therefore, this section exhibits an approximately 35° bias angle. This also verifies the reliability of the simulation model and the effectiveness of the selected sensor locations.

With the gradual increase in temperature from April to September, a slight increment in the strain of the tunnel section is observed. Specifically, the maximum tensile strain of section K107+043 is localized at the left spandrel, whereas the maximum compressive strain occurs at the right spandrel, reaching respective peaks of 110.4 µε and 245.48 µε within the 5-month period. Similarly, for section K107+240, the maximum tensile strain manifests at the left spandrel, with the maximum compressive strain concentrated at the right spandrel, peaking at 208.9 µε and 262.4 µε, respectively, over the same duration. Based on the stress analysis of the tunnel cross-section, it is determined that both monitoring sections exhibit bias pressure, indicating a left-side bias condition.

**Figure 12:**
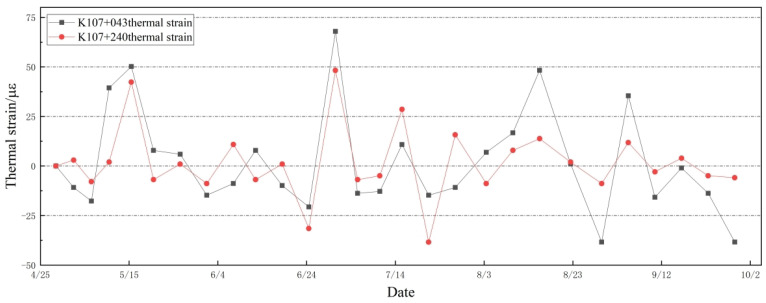
Thermal strain changes in two sections K107+043 and K107+240 over 5 months.

**Figure 13:**
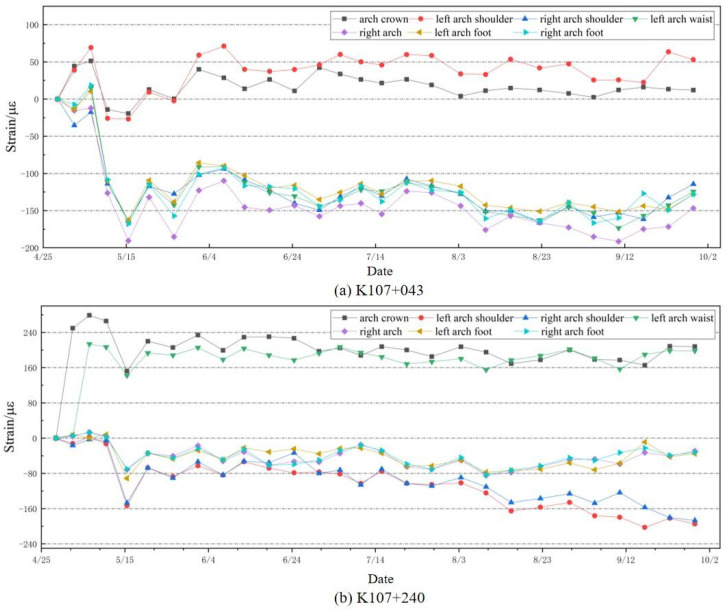
The applied stress–strain of K107+043 and K107+240 cross-sections during 5 months.

The monitoring system employs Equation (5) to demodulate thermal strain and applied stress–strain, as illustrated in Figure 12 and Figure 13, respectively. Throughout the temperature change process, the thermal apparent strain of the secondary lining undergoes significant variations. Both sections K107+043 and K107+240 exhibit an initial thermal strain of 0 µε, with the maximum thermal strains reaching 67.97 µε and 48.27 µε, respectively, occurring on the same day.

In Figure 13a, a notable fluctuation in applied stress–strain is observed from April to May, followed by a reduction in amplitude from June to September. In Figure 13b, evident fluctuations in applied stress–strain persist from April until mid-May, tapering off thereafter until September. The conspicuous fluctuations in monitored strain during the initial period suggest that disturbances from surrounding rocks render the secondary lining structure unstable. Conversely, as the amplitude of strain fluctuations gradually diminishes, it is indicative of the lining structure attaining a relatively stable state. Consequently, it becomes apparent that thermal strain becomes the primary contributor to secondary lining alterations once applied stress–strain fluctuations stabilize, whereas the pressure exerted by the surrounding rock on the lining structure is in a stable state.

## 5. Discussion and Conclusions

Excavation activities in tunnel engineering can disturb surrounding rocks, leading to structural deformations, fractures, and potential instability. Therefore, monitoring the lining is crucial for ensuring tunnel safety. This study proposes an integrated approach that combines simulation, structural deformation monitoring, and sensor-based surveillance to provide a reliable method for assessing tunnel engineering stability.

Geological data acquired from the exploration of the Deqin Tunnel formation were used to establish a tunnel cross-section simulation model. The model analyzed stress and displacement concentration areas of the tunnel lining under different bias angles, providing a theoretical basis for sensor layout and ensuring the validity of monitoring data. Additionally, the reliability and rationality of the model were verified through the analysis of lining monitoring data from actual projects. Subsequently, the displacement of the vault and surrounding areas of the Deqin tunnel cross-section lining structure was monitored and analyzed. Finally, FBG strain sensors were employed to monitor secondary lining strains. The key findings of this research are outlined below:

(1) Using geological data from the Deqin Tunnel formation, a tunnel cross-section simulation model was established to analyze stress and displacement concentration areas of the tunnel lining under different bias pressures. The simulation results indicate that under different bias angles, the maximum displacement, tensile stress, and compressive stress of the lining structure occur at different locations. As the bias angle of the mountain increases, the displacement concentration points and stress concentration points of the secondary lining structure shift accordingly. Therefore, simulation analysis can optimize the sensor deployment array, providing a theoretical basis for a more accurate assessment of tunnel stability.
(2) The vault and peripheral displacements of the tunnel have undergone a process of inward convergence and outward expansion. Through the monitoring and analysis of lining displacement, the stability of the tunnel can be evaluated in a short period, providing a theoretical basis for selecting sensor monitoring sections.
(3) The applied stress–strain of the secondary lining undergoes significant short-term fluctuations before stabilizing. Short-term excavation disturbances render the surrounding rock unstable initially, gradually transitioning to stability. Hence, continuous real-time monitoring of the lining over an extended duration is imperative. Such monitoring serves as a means to evaluate tunnel stability effectively.
(4) During the monitoring period, both tunnel lining sections experienced short-term disturbances from the surrounding rock. During this disturbance period, the maximum daily strain differences for the two sections were 126.87 µε and 209.38 µε, respectively. After the disturbance, the average daily strain differences in the applied stress–strain were 16.8 µε and 12.65 µε, respectively. Following a period of disturbance to the surrounding rock, the fluctuations in applied stress and strain gradually stabilized. Thus, the primary cause of strain in the secondary lining is thermal strain. This study combines simulation analysis and actual monitoring and proposes a method to simultaneously measure thermal strain and actual strain, providing a more comprehensive technical means and monitoring method for evaluating tunnel stability.

## Figures and Tables

**Table 1:** Calculation parameters of the materials.

Material Name	Elastic Modulus (E/GPa)	Density $\left({kg/m}^{3}\right)$	Poisson’s Ratio	Fiction Angle ($\Theta {/}^{{}^{\circ}}$)
Gravel	4	1.50 × $10^{3}$	0.43	25
Fault fracture zone	10	2.00 × $10^{3}$	0.38	20
Tunnel lining	30	2.50 × $10^{3}$	0.20	-

## Data Availability

Data are contained within the article.
